# Rapid evolution mitigates the ecological consequences of an invasive species (*Bythotrephes longimanus*) in lakes in Wisconsin

**DOI:** 10.1098/rspb.2017.0814

**Published:** 2017-07-05

**Authors:** Michael K. Gillis, Matthew R. Walsh

**Affiliations:** Department of Biology, University of Texas at Arlington, Arlington, TX 76019, USA

**Keywords:** rapid evolution, ecosystem services, invasive species, *Daphnia*, life history, predation

## Abstract

Invasive species have extensive negative consequences for biodiversity and ecosystem health. Novel species also drive contemporary evolution in many native populations, which could mitigate or amplify their impacts on ecosystems. The predatory zooplankton *Bythotrephes longimanus* invaded lakes in Wisconsin, USA, in 2009. This invasion caused precipitous declines in zooplankton prey (*Daphnia pulicaria*), with cascading impacts on ecosystem services (water clarity). Here, we tested the link between *Bythotrephes* invasion, evolution in *Daphnia* and post-invasion ecological dynamics using 15 years of long-term data in conjunction with comparative experiments. Invasion by *Bythotrephes* is associated with rapid increases in the body size of *Daphnia*. Laboratory experiments revealed that such shifts have a genetic component; third-generation laboratory-reared *Daphnia* from ‘invaded’ lakes are significantly larger and exhibit greater reproductive effort than individuals from ‘uninvaded’ lakes. This trajectory of evolution should accelerate *Daphnia* population growth and enhance population persistence. We tested this prediction by comparing analyses of long-term data with laboratory-based simulations, and show that rapid evolution in *Daphnia* is associated with increased population growth in invaded lakes.

## 1. Introduction

Invasive species are increasingly recognized as significant agents of global change [1–3] that pose threats to biodiversity [4,5]. This includes a growing recognition that invasive species alter community interactions and ecosystem functions, with cascading impacts on ecosystem services [6–8]. It is also now clear that anthropogenic forces (i.e. species invasions, climate change, artificial selection, etc.) can drive evolutionary changes in the traits of organisms within periods of years to decades [9–15]. Such rapid evolution provides a pathway to ecological processes [16]. Yet the extent to which contemporary adaptations may ameliorate the negative consequences of invasive species, including their impacts on ecosystem services, is unclear [17].

The spiny water flea (*Bythotrephes longimanus*; hereafter ‘*Bythotrephes*’) is a recent invader into many lakes in North America from its native Europe [18]. *Bythotrephes* is a dominant predator on herbivorous zooplankton, perhaps even accounting for a majority of zooplanktivory in some lakes [19]. As a result, invasions by *Bythotrephes* are associated with declines in zooplankton richness and biomass [20–22], with cascading impacts on phytoplankton communities [23,24]. *Bythotrephes* was first detected in two well-studied lakes in southern Wisconsin, USA, in 2009 (Lake Mendota and Lake Monona), causing a 60% reduction in the biomass of a key algal grazer (*Daphnia pulicaria*). *Daphnia pulicaria* support the local fishery [25] and maintain clear water via intense grazing on phytoplankton [26]. *Bythotrephes*-driven declines in the biomass of *Daphnia* are in turn associated with the degradation of a crucial ecosystem service, water clarity, of nearly 1 m (valued at a cost of US$140 million) [24]. Predator-mediated selection is a dominant driver of life-history evolution [27–31], and life-history evolution in *Daphnia* can alter community and ecosystem processes [32]. Thus, evolution in *Daphnia* in response to invasions by *Bythotrephes* may provide a means to mitigate or exacerbate the declines in ecosystem services that have occurred in Wisconsin.

Here, we used an ongoing synergy between long-term data in lakes in Wisconsin in conjunction with laboratory experiments to test the interplay between *Bythotrephes* invasion, evolution in *Daphnia* and post-invasion ecological dynamics. First, we leveraged 15 years of data from the North Temperate Lakes Long Term Ecological Research (NTL LTER) programme to evaluate phenotypic changes (i.e. body size) in *D. pulicaria* before and after invasions in Lake Mendota and Lake Monona. We compared these trends with phenotypic patterns in lakes that have not been invaded by *Bythotrephes*. Second, we evaluated the extent to which the observed phenotypic trends are explained by additional factors that are correlated with the arrival of *Bythotrephes* in Lake Mendota and Lake Monona. Third, we tested if invasion by *Bythotrephes* is associated with evolution in *Daphnia* by comparing the life-history traits of *Daphnia* from ‘invaded’ versus ‘uninvaded’ lakes after multiple generations of common garden rearing [33]. *Bythotrephes* have been shown to selectively prey upon large *D. pulicaria* [34]. Increased mortality targeted at large size classes favours the evolution of earlier maturation, smaller size and increased reproductive effort [27,28]. Therefore, we predict that invasion by *Bythotrephes* will be associated with reduced body size over time in the long-term data, and the evolution of earlier maturation, smaller size and increased reproductive output in the common garden experiments.

Our final goal was to test for an influence of *Daphnia* evolution on ecological processes using the known connection between life-history traits and population growth [35]. *Daphnia* in temperate lakes are found at low densities in the winter, before attaining peak abundances in the spring. However, *Bythotrephes* is not typically observed in the water column during this period of *Daphnia* population growth. This enabled us to quantify rates of *Daphnia* population growth before and after invasions in Lake Mendota and Lake Monona, in the absence of the confounding effects of *Bythotrephes* predation (using LTER data). We then compared these differences with simulations (based upon the common garden data) of population dynamics in ‘invaded’ versus ‘uninvaded’ lakes.

## 2. Material and methods

### (a) Long-term phenotypic trends

We evaluated shifts in the body size of *Daphnia* over time in two lakes that were invaded by *Bythotrephes* (Lake Mendota and Lake Monona) and five uninvaded lakes where *Bythotrephes* is absent (Allequash, Big Muskellunge, Crystal Bog, Fish and Trout Bog). These lakes are part of the North Temperate Lakes LTER. Data were obtained from the NTL LTER database (http://lter.limnology.wisc.edu) for 2000–2015 (datasets are cited in electronic supplementary material, table S1). This allows an evaluation of shifts in zooplankton size 8 years before the arrival of *Bythotrephes* (2000–2008) and 6 years after invasions (2009–2015), although data were not available for all years in the uninvaded lakes. We limited our analyses to data from June to November, as this spans the months that *Bythotrephes* and *Daphnia* are common in lakes. We also limited our analyses to samples in which more than one individual was measured. Because we were interested in shifts in adult body size, we removed all size estimates that were less than 1 mm. These values are less than half the average size at maturation for *D. pulicaria* (see ‘Common garden experiments’ below), and are therefore unlikely to be mature. All zooplankton data were obtained via a vertical tow at the deepest portion of each lake with an 80 µm mesh conical net. *Daphnia* body length was determined by photographing a subsample of individuals from each sample, and measuring the distance from the top of the head to the base of the tail spine via software stemming from the NTL LTER project (software.lter.limnology.wisc.edu).

We evaluated differences in *Daphnia* size before and after invasion by *Bythotrephes* using linear mixed models (via SPSS v. 23, IBM Corporation). We entered time period (pre-invasion, post-invasion), invasion status (invaded, uninvaded) and the ‘time period × invasion status' interaction as fixed effects. Lake (nested within status) was entered as a random effect. The unit of replication was the average size per year per lake (code is available in electronic supplementary material, appendix S1).

### (b) Ecological correlates of invasion

We obtained data on variables in Lake Mendota and Lake Monona from the NTL LTER database that may influence zooplankton size (electronic supplementary material, table S1). We collated yearly estimates from 2000 to 2015 for water temperature, duration of ice cover, Secchi depth, nutrients (nitrogen, phosphorus), planktivorous fish abundance and phytoplankton abundance. Physical parameters (temperature, Secchi depth) were collected at 1 m intervals every two weeks during the ice-free season. Nutrient levels were evaluated at the top and bottom of the epilimnion monthly during the ice-free period. Fish abundances were evaluated yearly. We focused our analyses on the density (catch per unit effort, CPUE) of bluegill (*Lepomis macrochirus*), which is a common planktivore in the dataset. We calculated the average CPUE from fish collected via beach seines, electrofishing and fyke nets. For the phytoplankton data, we were interested in temporal shifts in the biovolume of three common groups of phytoplankton (*Chlorophyta*, *Cyanobacteria* and *Bacillariophyta*). We limited these analyses to average epilimnetic values per sampling episode (defined as surface water layer where change in temperature is less than 1°C m^−1^).

For all parameters, we first evaluated trends over time via linear regressions in each lake. We then evaluated differences between the pre- and post-invasion period using general linear models with time period used as a categorical variable. The unit of replication for these parameters was the average value per year per lake. We then explored the influence of the ecological variables on *Daphnia* size using principal components regression [36]. Such an approach combines a principal components analysis (PCA) with multiple regression and permits a multivariate analysis of the significant predictors of *Daphnia* size, but eliminates concerns regarding multicollinearity. For each lake, we performed a PCA to evaluate the patterns of covariation among the variables. We retained all components with eigenvalues greater than 1 from the PCA. We then performed a multiple regression with *Daphnia* size entered as the dependent variable and components entered as independent variables.

### (c) Common garden experiments

We collected *D. pulicaria* from 10 lakes in Wisconsin that differ in the presence and absence of *Bythotrephes* (electronic supplementary material, table S2) in May 2016 via plankton tows (80 µm mesh net). To increase the likelihood that individuals sampled within a lake are genetically different, we performed greater than 10 plankton tows from distinct locations in each lake. We isolated live adult females from these samples (hereafter referred to as ‘clones’) and transported them to laboratory facilities at UTA. We isolated 63 clones of *D. pulicaria* from six lakes with *Bythotrephes* (Gile—12, Kegonsa—5, Mendota—20, Monona—15, Stormy—4, Waubesa—7) and 43 clones from four lakes that have not been invaded by *Bythotrephes* (Allequash—13, Beulah—5, Big Muskellunge—12, Rock—13). All individuals were cultured at 14°C (photoperiod 12 L : 12 D), slowly acclimated to COMBO media [37] and fed ample quantities of green algae (*Scenedesmus obliquus*; concentration: approx. 1.0 mg C l^−1^ d^−1^).

We reared all clones under common garden conditions for two generations prior to initiating the experiments. To establish the first laboratory generation, we collected four newly born (less than 12 h old) individuals from each parental clone and divided these individuals equally between two 90 ml jars containing COMBO medium and algae (concentration: 1.0 mg C l^−1^ d^−1^). These individuals were transferred to fresh media and algae every other day and were reared under the same conditions as the parents. Upon the release of the first clutch, all *Daphnia* were evaluated twice daily for the production of the second clutch. Offspring from this clutch were used to generate the second common garden generation using identical procedures as above.

We evaluated all populations for differences in life-history traits using third-generation laboratory-raised individuals. To begin the experiment, six newly born individuals were collected from the third clutch of each clone, individually placed in 90 ml jars, and randomly assigned to one of two treatments: (i) ‘no predator’ or (ii) ‘predator’. The ‘predator’ treatment contained media conditioned by fish chemical cues in generation one, while the ‘no predator’ treatment never received fish kairomones. These treatments assessed *Daphnia* from invaded versus uninvaded lakes for differences in sensitivity to predator cues. Kairomones were generated by collecting media conditioned by planktivorous fish daily from 7.5 l tanks containing redbreast sunfish (*Lepomis auritus*; average total length of fish = 50 mm). Each morning, media were removed from the aquaria and filtered using membrane filters (47 mm diameter, 0.45 µm mesh). The concentration of kairomones that was used in this experiment was 0.13 fish 1^−1^. Even though redbreast sunfish do not occur in lakes in Wisconsin, there is little evidence to suggest that *Daphnia* respond to fish cues in a species-specific manner. Each treatment was replicated 3× per clone (*n* = 106 clones × 2 treatments × 2 generations × 3 replicates = 1272 jars). All clones were transferred to fresh media and algae (and kairomones where appropriate) every other day throughout the experiment.

Beginning on day 5, all *Daphnia* were examined for the release of the first clutch into the brood chamber (i.e. maturation) 2× per day (at approx. 08.00 and 19.00). When the release of the first clutch was confirmed, age at maturation and clutch size was recorded, and each individual was photographed for estimates of size at maturation (using ImageJ). Size at maturation was measured as the distance from the top of the head to the base of the tail spine. All *Daphnia* were subsequently examined daily for the production of clutches 2–4. To initiate the second experimental generation, we collected newly born individuals from the second clutch of each jar and placed them into a new jar containing media and algae. The collection of life-history data in the second experimental generation parallels the procedures described above.

All dependent variables were analysed using liner mixed models implemented with restricted maximum-likelihood via SPSS v. 23 (IBM Corporation). We included invasion status, predator cue, generation and all interactions as fixed effects. Lake (nested within status) and clone (nested within lake) were included as random effects. When random effects were non-significant (*p* > 0.05), they were removed from the model and data were reanalysed without them. The data for age at maturation were ln-transformed and the data for clutch size were square-root transformed to improve normality and homogeneity of variances.

### (d) Population growth

To determine if evolution in *Daphnia* is associated with shifts in rates of population growth, we performed several analyses to synergize the long-term and experimental data. First, we combined estimates of age at maturation, clutch size and interclutch interval from the laboratory common garden data to calculate intrinsic rates of increase (*r*). *r* was calculated as: *r* = ln(*R*_0_)/*G*, where *R*_0_ is the net reproductive rate and *G* the generation time [35]. We tested for differences in *r* between invaded and uninvaded lakes via a linear mixed model (fixed effect: invasion status; random effect: lake). Second, we simulated changes in population size from the common garden data using the formula: *N_t_* = *N*_0_e*^rt^* (*N_t_* is the population size at time *t*, *N*_0_ the initial population size, *r* the intrinsic rate of increase and *t* the time in days). We calculated the duration of time required to reach the average peak abundance of *D. pulicaria* in Lake Mendota in the years following invasion by *Bythotrephes* (2009–2015). We used Lake Mendota as the reference lake because the negative consequences of *Bythotrephes* are well known in this lake [24]. We compared this value between invaded and uninvaded lakes with a linear mixed model (fixed: invasion status; random: lake).

Finally, we fitted exponential growth curves to the abundance data for *D. pulicaria* in Lake Mendota and Monona (from 2000 to 2015). For each year, we fitted exponential curves between the first winter sampling date and peak abundance attained each spring/summer. We excluded data for Lake Mendota in 2015 because there were no abundance estimates until late May (27 May 2015). We also excluded the data for Lake Monona in 2003 because the exponential model did not converge to the data and the resultant parameter estimate was an extreme value (greater than 3× length of box and whisker plot). We compared rates of increase between the pre- (2000–2008) and post-invasion (2009–2015) time periods via a two-way analysis of variance with time period (pre-, post-invasion), lake (Mendota, Monona) and the lake × time period interaction entered as fixed effects. We included the initial abundance of *Daphnia* (i.e. estimated population size of *Daphnia* on the first sampling date of each year) and the day of first sampling event as covariates to account for differences in starting conditions. We also tested for differences in the initial abundances of *Daphnia* between the pre- and post-invasion period via a two-way analysis of variance with time period and lake included as factors.

## 3. Results

### (a) Long-term phenotypic trends

**Figure 1.**
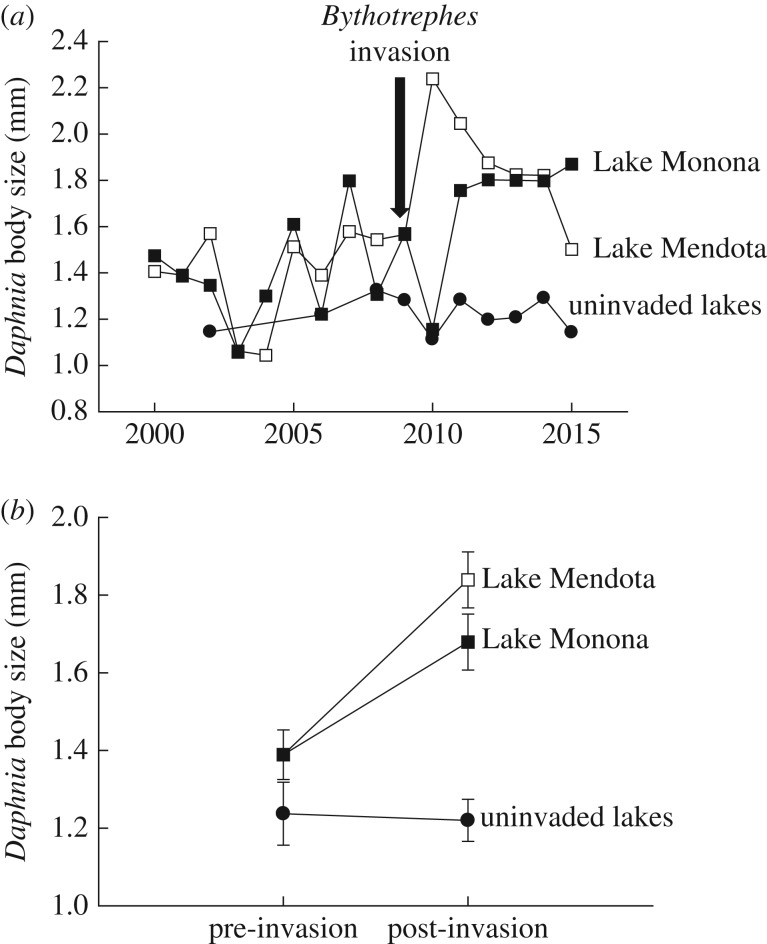
Temporal changes in *Daphnia* size. (*a*) Body size trends in *D. pulicaria* in Lake Mendota (open squares), Lake Monona (closed squares) and ‘uninvaded’ lakes (closed circles) from 2000 to 2015. (*b*) Variation in body size before and after invasions by *Bythotrephes* in invaded (Lake Mendota and Monona) and uninvaded lakes. ‘Pre-invasion’: 2000–2008. ‘Post-invasion’: 2009–2015. We observed a significant (*p* < 0.05) ‘time period × invasion’ interaction. *Daphnia* body size did not differ significantly in the pre-invasion period, but was significantly larger in the post-invasion period in invaded lakes. All data points are estimated marginal means and associated standard errors (±1.0 s.e.) generated by the linear mixed models.

Differences in body size between the ‘pre-’ and ‘post-invasion’ time period depended upon invasion status (i.e. invaded versus uninvaded) (invasion status × time period: *F*_1,54.1_ = 12.75, *p* = 0.001). Small differences in body size were observed between invaded and uninvaded lakes in the ‘pre-invasion’ time period; *Daphnia* from invaded lakes were 12% larger. We observed greater differences in the ‘post-invasion’ time period. *Daphnia* from invaded lakes were 44% larger than *Daphnia* from uninvaded lakes in the ‘post-invasion’ period (figure 1). The interaction was driven by phenotypic changes in invaded lakes; *Daphnia* in invaded lakes increased in size by 21–32% in the ‘post-’ versus ‘pre-invasion’ period. We detected no changes in *Daphnia* size between time periods in the uninvaded lakes. Overall, *Daphnia* were 28% larger in invaded versus uninvaded lakes (figure 1; invasion: *F*_1,3.7_ = 16.89, *p* = 0.017). *Daphnia* were also 13% larger in the post- versus pre-invasion period (time period: *F*_1,54.1_ = 10.56, *p* = 0.002). Differences among lakes within invasion status were not significant (Wald *Z* = 0.79, *p* = 0.43). We followed up the significant ‘invasion status × time period’ interaction with tests of simple main effects to evaluate differences in size between invaded versus uninvaded lakes independently in the pre- and post-invasion time periods. Because these tests made two additional comparisons, we used the *Bonferroni* correction to adjust our *p*-values and considered *p* < 0.025 as being ‘significant’ (0.05/2 = 0.025). These tests revealed non-significant differences between invaded and uninvaded lakes in the pre-invasion period (*F*_1,23_ = 2.89, *p* = 0.1), but significant differences in the post-invasion period (*F*_1,35_ = 57.6, *p* < 0.001).

### (b) Ecological correlates of invasion

Linear regressions revealed significant (*p* < 0.05) declines in Secchi depth in both lakes (electronic supplementary material, figure S1). All other trends were not significant (*p* > 0.05), save for a significant increase in the biovolume of *Bacillariophyta* in Lake Mendota (electronic supplementary material, figure S1). General linear models revealed non-significant (*p* > 0.05) differences between the pre- and post-invasion periods for all variables, except for a significant decline in Secchi depth in the post-invasion period (approx. 26% reduction; electronic supplementary material, table S3 and figure S2). A PCA that included all of the physical, chemical and biological parameters retained four components that explained 78.2% of the variance (variance explained: PC1 = 31.51%, PC2 = 20.15%, PC3 = 14.22% and PC4 = 12.29%; electronic supplementary material, figure S3). The subsequent results of a principal components regression were non-significant (*r*^2^ = 0.045; *F*_4,23_ = 0.27, *p* = 0.9; electronic supplementary material, table S4).

### (c) Common garden experiments

**Figure 2.**
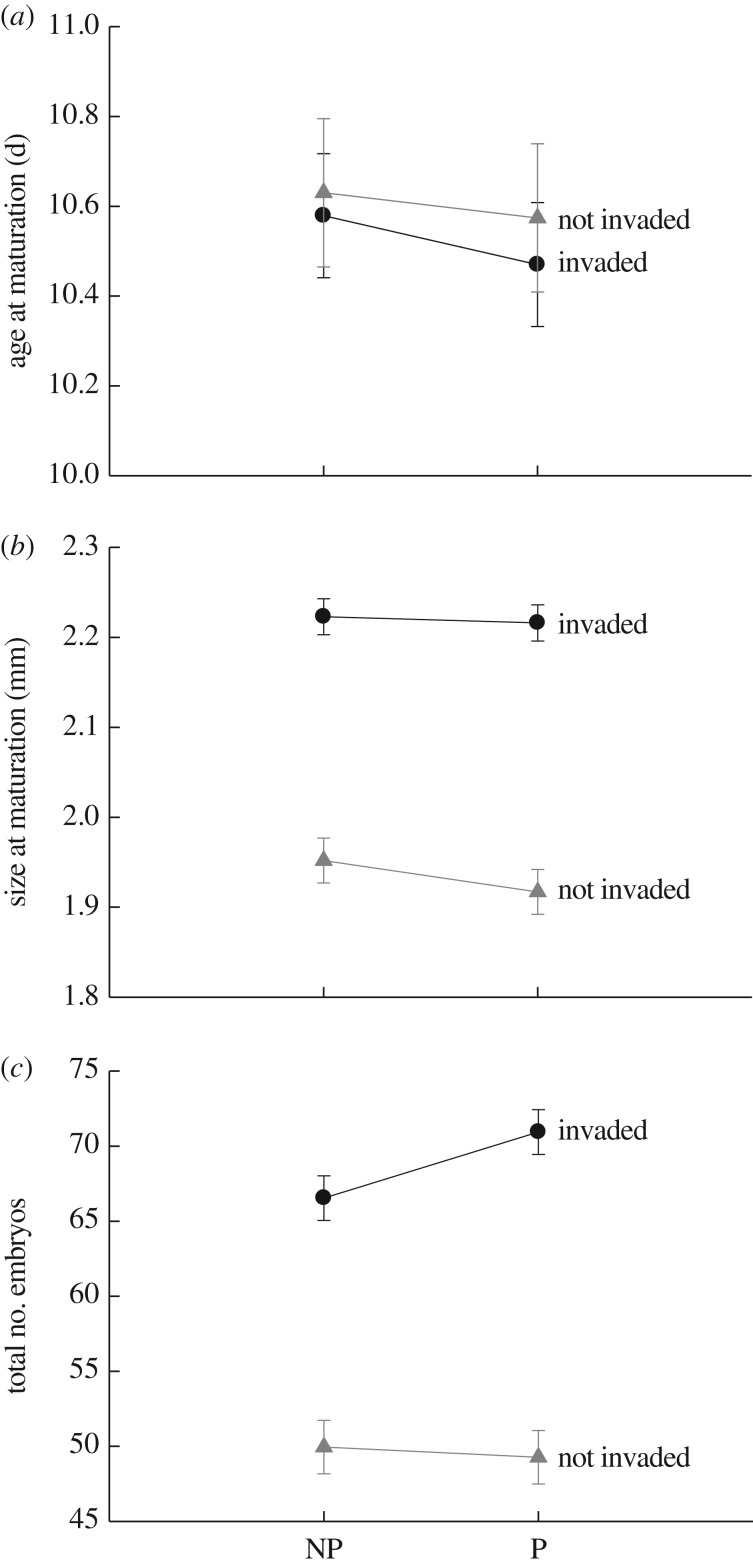
Variation in life-history traits and trait plasticity between invaded and uninvaded lakes. (*a*) Age at maturation, (*b*) size at maturation and (*c*) number of embryos. Black circles, invaded lakes; grey triangles, uninvaded lakes. NP, ‘no predator’ treatment; P, ‘predator’ treatment. We observed significant (*p* < 0.05) differences between invaded and uninvaded lakes for size at maturation and clutch size. Differences in age at maturation were not significant (*p* > 0.05). We observed significant ‘predator × invasion’ interactions for size at maturation and clutch size. All data points are estimated marginal means and associated standard errors (±1.0 s.e.) generated by the linear mixed models.

We observed significant differences in *Daphnia* from invaded versus uninvaded lakes for size at maturation and clutch size (table 1 and figure 2; electronic supplementary material, table S5). *Daphnia* from invaded lakes produced 39% more offspring (for clutch 1–4) and were 15% larger at maturation than *Daphnia* from uninvaded lakes. Differences in age at maturation were not significant (table 1). We also observed significant interactions between invasion status and predator cues for size at maturation and clutch size, but not age at maturation (table 1 and figure 2). Here, the differences in the traits of *Daphnia* between invaded and uninvaded lakes were slightly larger in the presence than absence of fish kairomones. *Daphnia* from invaded lakes matured at a size that was 14% larger and produced 33% more offspring than *Daphnia* from uninvaded lakes in the absence of predator cues, and such differences increased to 16% and 44% in the presence of predator cues, respectively (figure 2). There was little evidence for transgenerational responses to predator cues. All ‘predator × generation’ and ‘predator × invasion × generation’ interactions were not significant (table 1).

**Table 1.** Analyses of *Daphnia* characteristics between invaded and uninvaded lakes. Differences in *Daphnia* traits were analysed using general linear models with invasion status, predator cue and generation entered as fixed effects. Entries for the fixed effects are *F*-statistics, while entries for the random effects are Wald-*Z* values from a likelihood ratio test. Italics indicate significant results (*p* < 0.05).

factor	d.f.	age at maturation		no. embryos		size at maturation	
		*F*	*p*-value	*F*	*p*-value	*F*	*p*-value
fixed effects							
generation	1	*79.4 (973)*	*<0.001*	*190.4 (951)*	*<0.001*	*552.3 (990)*	*<0.001*
invasion status	1	1.26 (101)	0.27	*73.6 (103)*	*<0.001*	*82.8 (104)*	*<0.001*
predator	1	0.4 (972)	0.54	*10.1 (953)*	*0.002*	*10.3 (990)*	*0.001*
generation × invasion	1	3.16 (973)	0.08	*50.8 (951)*	*<0.001*	*29.5 (990)*	*<0.001*
generation × predator	1	0.01 (968)	0.92	2.42 (950)	0.12	0 (990)	0.98
invasion × predator	1	0.02 (972)	0.88	*22.8 (953)*	*<0.001*	*4.3 (990)*	*0.038*
generation × invasion × predation	1	0.03 (968)	0.86	0.67 (950)	0.41	14 (988)	0.24
random effects							
lake (invasion)	1	1.8	0.072	1.66	0.096	1.63	0.1
clone (lake)	1	*6.2*	*<0.001*	*6.81*	*<0.001*	*6.86*	*<0.001*

The traits of *Daphnia* differed significantly between generation 1 and 2 of the common garden experiment. *Daphnia* matured 6% later, were 8% smaller and produced 12% fewer embryos in generation 2 versus 1 (table 1; electronic supplementary material, table S5). We also observed a significant generation × invasion interaction for size at maturation and clutch size (table 1). *Daphnia* from lakes with *Bythotrephes* were consistently larger at maturation and exhibited greater reproductive outputs, but such differences were larger in generation 2 versus generation 1. For instance, *Daphnia* from invaded lakes produced 30% more offspring than *Daphnia* from uninvaded lakes in generation 1 and such differences increased to 49% in generation 2 (average clutch size: invaded gen. 1 ± s.e. = 70.52 ± 1.48, uninvaded gen. 1 ± s.e. = 54.35 ± 1.78, invaded gen. 2 ± s.e. = 67.0 ± 1.5, uninvaded gen. 2 ± s.e. = 44.87 ± 1.79). The trends were similar for the significant generation × invasion interaction for size at maturation (average size at maturation: invaded gen. 1 ± s.e. = 2.28 ± 0.02, uninvaded gen. 1 ± s.e. = 2.03 ± 0.025, invaded gen. 2 ± s.e. = 2.16 ± 0.021, uninvaded gen. 2 ± s.e. = 1.84 ± 0.025).

### (d) Population growth

**Figure 3.**
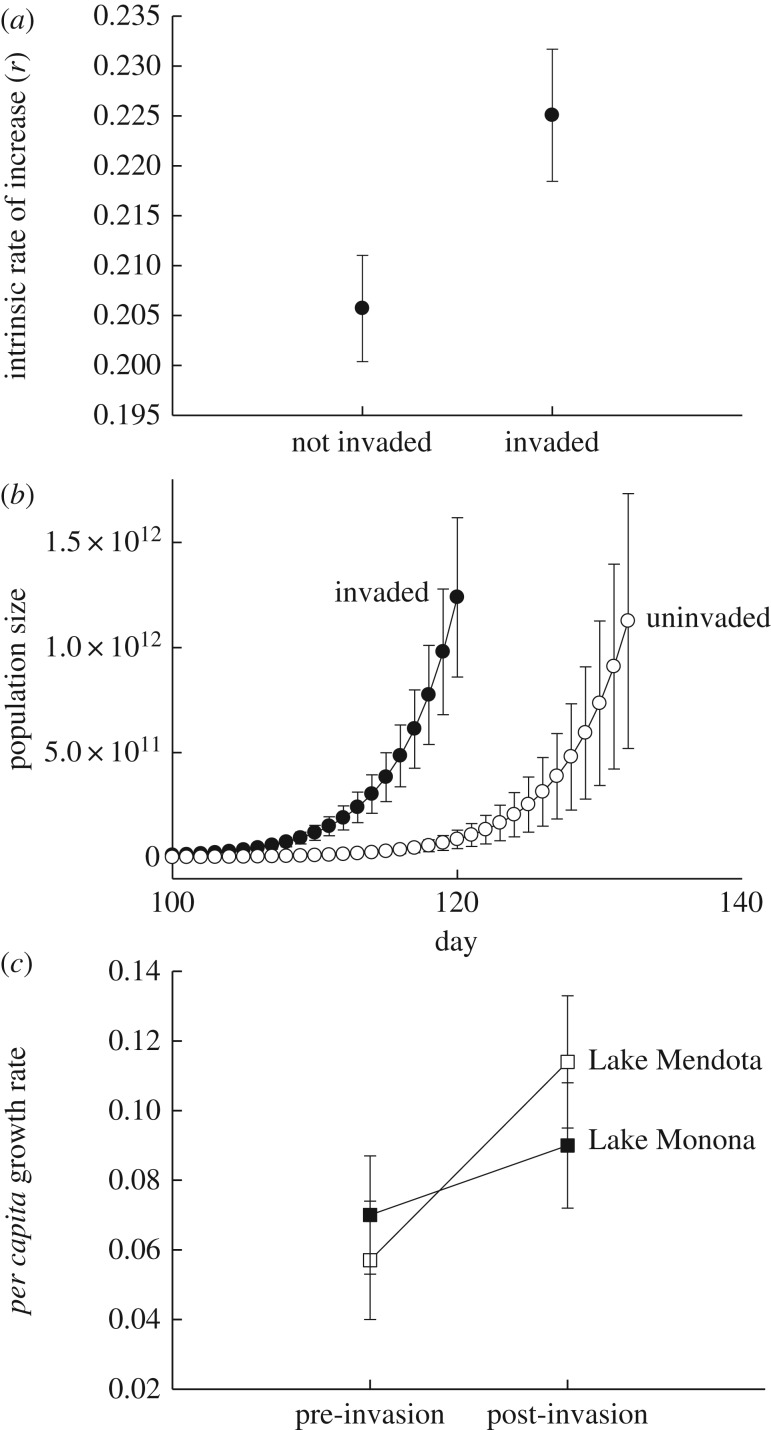
Invasion by *Bythotrephes* is associated with shifts in *Daphnia* population growth rates. (*a*) Intrinsic rate of increase between invaded and uninvaded lakes generated by the common garden data. Differences in *r* were marginally non-significant. (*b*) Average duration of time for *Daphnia* from invaded (closed circles) and uninvaded (open circles) lakes to reach peak densities observed in Lake Mendota after invasion. These differences were marginally non-significant (*p* < 0.1). (*c*) *Per capita* growth rates in Lake Mendota and Monona before and after invasion by *Bythotrephes*. Pre-invasion: 2000–2008, post-invasion: 2009–2015. Open squares, Lake Mendota; closed squares, Lake Monona. Differences in the initial abundances of *Daphnia* were not significant (*p* > 0.05) between the pre- and post-invasion time periods. We observed significant (*p* < 0.05) differences in population growth between the pre- and post-invasion time periods. Error = ±1.0 s.e.

Differences in rates of intrinsic increase between *Daphnia* from invaded and uninvaded lakes from the common garden data were marginally non-significant (*F*_1,8_ = 4.36, *p* = 0.071); *Daphnia* from invaded lakes exhibited an *r* that was 10% greater than *Daphnia* from uninvaded lakes (figure 3). We used these data to calculate the duration of time required to attain average peak abundances that are now observed in Lake Mendota. *Daphnia* from invaded lakes exceed natural peak abundances approximately two weeks earlier than *Daphnia* from uninvaded lakes (figure 3). These differences in time to peak abundance were marginally non-significant (*F*_1,8_ = 4.3, *p* = 0.072).

We observed significant differences in rates of *Daphnia* population growth in Lake Mendota and Lake Monona between the pre- and post-invasion time period (*F*_1,24_ = 4.42, *p* = 0.046) (figure 3). Rates of increase were approximately 62% higher in the post- versus pre-invasion time period in both lakes. Growth rates did not differ between lakes (*F*_1,24_ = 0.09, *p* = 0.77). The lake × time period interaction was also not significant (*F*_1,24_ = 1.05, *p* = 0.32). The initial abundances of *Daphnia* (i.e. estimated population size of *Daphnia* on the first sampling date of each year) did not differ significantly between time periods (time period: *F*_1,25_ = 0.0, *p* = 0.99; initial density in individuals m^−2^: pre-invasion = 11 223.1 ± 4816, post-invasion = 11 340.1 ± 5360) or lakes (*F*_1,25_ = 0.57, *p* = 0.46), nor did initial densities depend upon combined effects of lake and time (lake × time period: *F*_1,25_ = 0.99, *p* = 0.33).

## 4. Discussion

Our results show that invasion by the invertebrate predator, *Bythotrephes*, is correlated with rapid phenotypic shifts in *Daphnia* in lakes in Wisconsin (figure 1). Prior to invasions, the average body sizes of *D. pulicaria* in Lake Mendota and Lake Monona did not differ significantly from individuals in lakes where *Bythotrephes* is absent. However, *Daphnia* in the ‘invaded’ lakes are now over 40% larger than individuals in ‘uninvaded’ lakes. These differences are due to recent phenotypic shifts in the invaded lakes. The size of *D. pulicaria* in Lake Mendota and Lake Monona has increased by more than 25% since 2009, but has not changed, on average, in uninvaded lakes (figure 1). Subsequent comparisons between contemporary populations that differ in the presence and absence of *Bythotrephes* showed that third-generation laboratory-reared *Daphnia* from invaded lakes were larger at maturation and exhibit a greater investment in reproduction than individuals from uninvaded lakes (figure 2). These results raise three important questions. (i) What ecological mechanisms underlie recent life-history shifts in *Daphnia* in invaded lakes? (ii) To what extent do these reflect genetic changes in response to invasions by *Bythotrephes*? (iii) Is there evidence for an ecological importance of evolution?

### (a) Mechanism(s) of phenotypic change

We analysed the temporal patterns of biotic and abiotic parameters in Lake Mendota and Monona to determine if the arrival of *Bythotrephes* covaried with other ecological changes that may explain shifts in *Daphnia* size. Water clarity has declined significantly in both lakes (electronic supplementary material, figures S1 and S2), which is consistent with known indirect effects of *Bythotrephes* predation [24]. Yet we did not observe significant changes in other variables, such as temperature or planktivorous fish, that may influence *Daphnia* size (electronic supplementary material, figures S1 and S2). The abundances of diatoms (*Bacillariophyta*) significantly increased over time, but only in Lake Mendota. Such data argue that the ecological consequences of *Bythotrephes*, and not correlated shifts in other components of the environment, underlie the observed changes in *Daphnia* body size. As these phenotypic shifts occurred exclusively in invaded lakes (figure 1), and are matched by life-history patterns observed in the laboratory (figure 2), we conclude that invasions by *Bythotrephes* have driven rapid (less than 7 years) phenotypic change in *Daphnia*.

Given the intensity of *Bythotrephes* predation documented in this and other systems [19,24], along with their preference to consume the largest size-classes of *D. pulicaria* [34], we predicted that *Bythotrephes* would select for smaller *Daphnia*. Instead, we observed shifts in the opposite direction. One explanation could be that *Daphnia* exceed the gape of *Bythotrephes*. However, the sizes of *Daphnia* reported in this study (approx. 2.0 mm) are within the feeding range of *Bythotrephes* found in other systems [34], and the average length of *Bythotrephes* in Lake Mendota is comparable with estimates reported in other studies [38]. Thus, direct predation by *Bythotrephes* may not offer a strong explanation for recent phenotypic changes in *Daphnia*. In addition to direct consequences of invasions, *Bythotrephes*-driven declines in the biomass of *Daphnia* have led to increased phytoplankton [24]. This is important because indirect effects of predation can also exert selection on life histories [29], and higher food availability selects for larger body sizes [39]. Evolutionary responses to indirect effects of predators can also outweigh impacts from direct consumption [40,41]. As the trajectory of phenotypic change observed here is consistent with aspects of theory that incorporates changes in resources from predation [42], we hypothesize that the indirect effects of *Bythotrephes* are important drivers of life-history modifications in this system.

However, it is important to note that *Daphnia* respond to the presence of *Bythotrephes* by migrating to deeper depths [43,44]. This is because the risk of predation declines with depth. Yet such areas are also lower in resources. These behavioural modifications could limit selection mediated by increased food availability. We also cannot eliminate a role for interspecific competition in the life-history changes documented in this study. *Bythotrephes* does not uniformly cause declines in all species of zooplankton. For instance, the abundances of *Daphnia mendotae* may not decline following *Bythotrephes* invasions [45], and did not decline until recently in Lake Mendota [24]. The manner in which the contrasting negative consequences of *Bythotrephes* predation alters competitive interactions, and potentially selection due to competition is unclear. It is also interesting to note that *Daphnia* populations in temperate lakes re-establish each spring from resting eggs found in lake sediment [46]. This seasonal pulse probably encompasses genotypes from multiple years, including genotypes that have never been exposed to *Bythotrephes*. Yet we observed consistent shifts in phenotype over time (figure 1), and consistent differences between populations (figure 2), despite likely ongoing input from historic propagules.

### (b) Genetic versus non-genetic changes

A key question is whether the shifts in *Daphnia* size in invaded lakes (figure 1) are heritable and represent evolutionary responses to predation by *Bythotrephes*. We tested this hypothesis by rearing *Daphnia* from invaded and uninvaded lakes for multiple generations in the laboratory to determine if the phenotypic trends are maintained in a common environment. These comparisons revealed strong genetically based differences in life-history traits between lakes with and without *Bythotrephes* (figure 2). One potential shortcoming of our laboratory experiments is that the focal lakes differ in additional variables that may influence the traits of *Daphnia*. Most notably, invaded lakes are more eutrophic than uninvaded lakes (electronic supplementary material, table S2). Differences in resource availability can influence *Daphnia* evolution [46].

However, there are several indications that the ecological impacts of *Bythotrephes* invasions, and not confounding differences among our focal lakes, underlie the trait differences revealed in the laboratory. First, there is a high degree of overlap between the lakes used in our long-term and experimental analyses. Over 50% of the clones used in our experiment come from LTER lakes that we evaluated for phenotypic changes over time. If pre-existing ecological variation among lakes is the cause of life-history divergence observed in the laboratory, we expected *Daphnia* in invaded lakes to differ from individuals in uninvaded lakes during the ‘pre-invasion’ period. Instead, we observed small differences in the body sizes of *Daphnia* from invaded and uninvaded lakes prior to 2009 (figure 1). Subsequent increases in body size in invaded lakes are correlated with invasions by *Bythotrephes* and declines in Secchi depth, but other factors that influence the size of *Daphnia* remained stable during this period (electronic supplementary material, figures S1 and S2). Furthermore, two of our focal lakes in northern Wisconsin that have been invaded by *Bythotrephes* and were included in the common garden experiments (Stormy, Gile) have historically been less productive than the uninvaded lakes used in the experiment (electronic supplementary material, table S2). However, *Daphnia* from these ‘low productivity’ invaded lakes attained a larger size at maturation and produced more offspring than individuals from uninvaded lakes. These observations suggest that historical differences among lakes are less important in explaining trait differences observed in the laboratory, compared with recent ecological changes that coincided with the arrival of *Bythotrephes*.

We also evaluated our focal populations for differences in phenotypic plasticity, because variation in predation regimes can alter selection on plasticity [33], and this may have ecological ramifications [47]. Each summer, *Daphnia* experience abrupt shifts in *Bythotrephes* predation in invaded lakes [24]. Such predator–prey dynamics are expected to favour enhanced plasticity [48,49]. However, it remains unclear how *Daphnia* modify life-history traits in response to *Bythotrephes* versus fish (but see [44]). Because we used fish kairomones to elicit phenotypic responses in our experiment, we refrained from making *a priori* predictions. We observed small but significant differences in predator-induced plasticity (though not transgenerational plasticity) in *Daphnia* from invaded and uninvaded lakes (figure 2). The direction of the changes follows overall life-history patterns observed between the invaded and uninvaded lakes. *Daphnia* from invaded lakes did not adjust their body size in the presence of fish kairomones, while *Daphnia* from uninvaded lakes showed reduced size after exposure to predator cues. This response to planktivorous fish is common [33]. Individuals from invaded lakes also responded to fish cues by elevating the size of their clutches to a greater extent than *Daphnia* from uninvaded lakes. Because these differences in plasticity are well matched to the overall trajectory of life-history evolution observed in invaded lakes (figure 2), they could represent adaptive responses to invasions by *Bythotrephes*. However, we advocate that additional experiments, using *Bythotrephes*, are needed to test this hypothesis.

### (c) Eco-evolutionary consequences of *Bythotrephes*

Invasion by *Bythotrephes* in Wisconsin has driven declines in the biomass of *Daphnia*, with cascading reductions in water clarity [24]. Given the importance of *Daphnia* grazing for regulating phytoplankton abundance [50,51], the evolutionary changes documented in this study may counteract this loss of ecosystem services by enhancing *Daphnia* persistence. For example, the evolution of increased reproductive outputs (figure 2) may boost population growth rates and work to offset increased predation by *Bythotrephes* [35]. If evolution due to invasion by *Bythotrephes* provides a pathway to ecology, we predicted that rates of *Daphnia* population growth will be faster in invaded versus uninvaded lakes, as well as in the ‘post-’ versus ‘pre-invasion’ period (in invaded lakes).

We found parallel shifts in population growth in the laboratory and field (figure 3). Our common garden data show that *Daphnia* from invaded lakes exhibited an *r* that was 10% higher than uninvaded lakes, and have the potential to attain peak levels of abundance observed naturally in Lake Mendota approximately two weeks earlier than *Daphnia* from uninvaded lakes. Such shifts are important because the earliest that *Bythotrephes* is typically detected in the water column is in early June. Faster population growth will allow *Daphnia* to attain higher densities prior to the arrival of *Bythotrephes* and offer greater resilience to this seasonal pulse of predation. Further, our field-based data showed that invasion by *Bythotrephes* is associated with a greater than 60% increase in *r* in the years following invasion (figure 3). These differences in growth are not due to differences in the initial densities of *Daphnia*. Such results collectively illustrate a connection between life-history evolution and population dynamics in lakes, and argue that the ecological consequences of *Bythotrephes* would be more pronounced in the absence of evolution.

Invasive species have extensive evolutionary consequences for native species [11–13]. This includes shifts to novel hosts/resources [52,53], responses to exotic competitors [54,55] and invasive predators driving evolution in native prey [56,57]. However, the ecological significance of adaptive responses to novel community members, particularly in regard to the provision of ecosystem services, has received less attention [17]. Here, we show that contemporary evolution during aquatic invasions influences the population dynamics of native zooplankton (*Daphnia*). Such effects are detectable at the whole-lake scale, and could be mitigating the impacts of an invader (*B. longimanus*) on a valuable ecosystem service (water clarity). Our results highlight evolution as a factor in both ecological and socio-economic outcomes of anthropogenic change.

## Supplementary Material

Supplementary figures

## Supplementary Material

Supplementary tables

## Supplementary Material

Appendix S1

## Data accessibility

LTER datasets are cited as electronic supplementary material, table S1. Long-term data can be freely accessed through the following website: http://lter.limnology.wisc.edu. All data used in the analyses, including data stemming from the common garden experiment, are deposited in Dryad: http://dx.doi.org/10.5061/dryad.qh47m [58].
