# Supplementary material for: Rapid evolution mitigates the ecological consequences of an invasive species (Bythotrephes longimanus) in lakes in Wisconsin
Source: Proc Biol Sci. 2017 Jul 5;284(1858):20170814. doi: 10.1098/rspb.2017.0814 (PMC5524501; doi:10.1098/rspb.2017.0814)
Supplement: Supplementary figures [file rspb20170814supp1.docx]

**Fig. S1.** Shifts in abiotic and biotic variables from 2000-2015 in Lake Mendota (ME) and Lake Monona (MO). (A) water temperature, (B) duration of ice cover, (C) Secchi depth, (D) phosphorus, (E) nitrogen, (F) bluegill abundance, (G) chlorophyta biovolume, (H) cyanobacteria biovolume, (I) bacillariophyta biovolume. Closed diamonds - Lake Mendota, Grey triangles – Lake Monona. Linear regressions revealed significant (p < 0.05) declines in Secchi depth over time.

**(A)**

**(B)**

**(C)**

ME: r = 0.35, p = 0.18
MO: r = 0.02, p = 0.95

ME: r = -0.71, p = 0.002
MO: r = -0.54, p = 0.031

ME: r = 0.09, p = 0.75
MO: r = 0.18, p = 0.5

**(D)**

**(E)**

**(F)**

ME: r = 0.12, p = 0.65
MO: r = -0.19, p = 0.48

ME: r = -0.12, p = 0.67
MO: r = 0.03, p = 0.91

ME: r = -0.11, p = 0.69
MO: r = -0.24, p = 0.41

**(G)**

**(H)**

**(I)**

ME: r = 0.59, p = 0.02
MO: r = -0.33, p = 0.25

ME: r = -0.17, p = 0.54
MO: r = -0.01, p = 0.99

ME: r = 0.08, p = 0.77
MO: r = 0.18, p = 0.56

**Fig. S2.** Variation in abiotic and biotic variables before (2000-2008) and after (2009-2015) invasion by *Bythotrephes* in Lake Mendota (closed diamonds) and Lake Monona (grey triangles). (A) water temperature, (B) duration of ice cover, (C) Secchi depth, (D) phosphorus, (E) nitrogen, (F) bluegill abundance, (G) chlorophyta biovolume, (H) cyanobacteria biovolume, (I) bacillariophyta biovolume. General linear models revealed significant (p < 0.05) declines in Secchi depth in the ‘post-invasion’ time period.

**(A)**

**(B)**

**(C)**

**(D)**

**(E)**

**(F)**

**(I)**

**(H)**

**(G)**

**Fig. S3.** Bivariate plot between principal component 1 and principal component 2. Each data point represents the factor loading per variable.

**Bluegill**

**Cyanobacteria**

**Water temperature**

**Ice duration**

**Bacillariophyta**

**Chlorophyta**

**Phosphorus**

**Nitrogen**

**Secchi**
