# Supplementary material for: Rapid evolution mitigates the ecological consequences of an invasive species (Bythotrephes longimanus) in lakes in Wisconsin
Source: Proc Biol Sci. 2017 Jul 5;284(1858):20170814. doi: 10.1098/rspb.2017.0814 (PMC5524501; doi:10.1098/rspb.2017.0814)
Supplement: Supplementary tables [file rspb20170814supp2.docx]

| **Variable** | **Dataset(s)** |
| --- | --- |
| Body size – *Daphnia pulicaria* | Center for Limnology; NTL LTER (2011): *North Temperate Lakes LTER: Zooplankton - Madison Lakes Area 1997 - current.* Center for Limnology, NTL LTER, Long Term Ecological Research Network. http://dx.doi.org/10.6073/pasta/f61e22c8f7a5a7832dae63cc02443e60  Center for Limnology; NTL LTER (1983): *North Temperate Lakes LTER: Zooplankton - Trout Lake Area 1982 - current.* Center for Limnology; NTL LTER; Long Term Ecological Research Network. http://dx.doi.org/10.6073/pasta/c866e3663bae76388f63233a5fdfb3d4 |
| Temperature | Center for Limnology; NTL LTER (1996): *North Temperate Lakes LTER: Ice Duration - Madison Lakes Area 1853 - current.* Center for Limnology; NTL LTER; Long Term Ecological Research Network. http://dx.doi.org/10.6073/pasta/9020edd8a00270724771421c9326fb33 |
| Ice Duration | Center for Limnology; NTL LTER (1996): *North Temperate Lakes LTER: Ice Duration - Madison Lakes Area 1853 - current.* Center for Limnology; NTL LTER; Long Term Ecological Research Network. http://dx.doi.org/10.6073/pasta/9020edd8a00270724771421c9326fb33 |
| Secchi Depth | Center for Limnology; NTL LTER (2012): *North Temperate Lakes LTER: Secchi Disk Depth; Other Auxiliary Base Crew Sample Data 1981 - current.* Center for Limnology; NTL LTER; Long Term Ecological Research Network. http://dx.doi.org/10.6073/pasta/1d2288f1c1824e2d402b56be0bba4b5a |
| Nutrients | Center for Limnology; NTL LTER (2012): *North Temperate Lakes LTER: Chemical Limnology of Primary Study Lakes: Nutrients, pH and Carbon 1981 - current.* Center for Limnology; NTL LTER; Long Term Ecological Research Network. <http://dx.doi.org/10.6073/pasta/3e5b0167913b81b58a0eb1596a3fba9d> |
| Fish Abundance – *Lepomis macrochirus* | Lead PI, NTL; Magnuson, John; Carpenter, Stephen; Stanley, Emily (2010-09-20): *North Temperate Lakes LTER: Fish Abundance 1981 - current.* Long Term Ecological Research Network. <http://dx.doi.org/10.6073/pasta/8c1ba9aab5724e9dc2ce7e3950302679> |
| Phytoplankton Abundance | Center for Limnology; NTL LTER (2011): *North Temperate Lakes LTER: Phytoplankton - Madison Lakes Area 1995 - current.* Center for Limnology; NTL LTER; Long Term Ecological Research Network. <http://dx.doi.org/10.6073/pasta/0ad6a347ba1e95bef61308e548ecb00d> |
| *Daphnia* *pulicaria* Abundance | Center for Limnology; NTL LTER (2011): *North Temperate Lakes LTER: Zooplankton - Madison Lakes Area 1997 - current.* Center for Limnology, NTL LTER, Long Term Ecological Research Network. http://dx.doi.org/10.6073/pasta/f61e22c8f7a5a7832dae63cc02443e60 |

**Table S1.** Variables analyzed and associated datasets available from the North Temperate Lakes LTER ([http://lter.limnology.wisc.edu](http://lter.limnology.wisc.edu/)).

**Table S2. Lake Properties.** Physical and biotic features of the focal lakes. ‘Year invaded’ denotes the first appearance of *Bythotrephes*. ‘F-test’ reflects the results of a one-way analysis of variance between the lakes that have and have not been invaded by *Bythotrephes*. These lakes do not differ significantly in depth, surface area, and total phosphorus. NS – not significant (p > 0.05).

**Table S3.** Analyses for ecological variables that potentially correlate with invasion by *Bythotrephes*. All parameters were analyzed using general linear models with lake (Mendota, Monona), time period (pre-, post-invasion), and the lake x time period interaction entered as fixed effects.

**Table S4.** Principal components regression results. ‘β coefficient’ displays the parameter values stemming from the multiple regression for each components. ‘t-statistic’ displays the t-value and significance level for each coefficient. NS: p > 0.05.

|  |  | |
| --- | --- | --- |
|  |  |  |
| **Component** | **β Coefficient** | **t-statistic** |
|  |  |  |
| **1** | **-0.15** | **-0.74NS** |
|  |  |  |
| **2** | **0.11** | **0.53NS** |
|  |  |  |
| **3** | **-0.09** | **-0.45NS** |
|  |  |  |
| **4** | **0.04** | **-0.2NS** |

**Table S5.** Mean trait values for each lake for experiments with *Daphnia* *pulicaria*. The parentheses following each entry represent the ±1.0 s.e.
