# Supplementary material for: Rapid evolution mitigates the ecological consequences of an invasive species (Bythotrephes longimanus) in lakes in Wisconsin
Source: Proc Biol Sci. 2017 Jul 5;284(1858):20170814. doi: 10.1098/rspb.2017.0814 (PMC5524501; doi:10.1098/rspb.2017.0814)
Supplement: Appendix S1 [file rspb20170814supp3.docx]

**Appendix S1. SPSS code for all analyses.**

**1. Phenotypic trends from the long-term data. These analyses tested for shifts in body size over time in lakes that have and have not been invaded by Bythotrephes.**

MIXED Length BY Lake Status TimePeriod

/CRITERIA=CIN(95) MXITER(100) MXSTEP(10) SCORING(1) SINGULAR(0.000000000001) HCONVERGE(0,

ABSOLUTE) LCONVERGE(0, ABSOLUTE) PCONVERGE(0.000001, ABSOLUTE)

/FIXED=Status TimePeriod Status*TimePeriod | SSTYPE(3)

/METHOD=REML

/RANDOM=Lake(Status) | COVTYPE(VC)

/EMMEANS=TABLES(Status) .

**2. We performed analyses to test for ecological correlates of invasion. We tested for shifts in several variables as a function of time. Below we provide coding for these correlational analyses for one of the variables.**

NEW FILE.

DATASET NAME DataSet7 WINDOW=FRONT.

CORRELATIONS

/VARIABLES=VAR00001 VAR00002

/PRINT=TWOTAIL NOSIG

/MISSING=PAIRWISE.

**We also tested for differences in these variables between the pre- and post-invasion time period using general linear models. Below we provide the code for one such analysis.**

DATASET NAME DataSet8 WINDOW=FRONT.

UNIANOVA Watertemp BY Time_period Lake

/METHOD=SSTYPE(3)

/INTERCEPT=INCLUDE

/CRITERIA=ALPHA(0.05)

/DESIGN=Time_period Lake Time_period*Lake.

**We performed a principal components regression analysis using these correlated ecological variables. Below is the code for the PCA.**

FACTOR

/VARIABLES Watertemp Unfilt_P TotalN Sechidepth lnchl lncyano lnbacil Iceduration bluegillcpue

/MISSING LISTWISE

/ANALYSIS Watertemp Unfilt_P TotalN Sechidepth lnchl lncyano lnbacil Iceduration bluegillcpue

/PRINT INITIAL EXTRACTION ROTATION FSCORE

/CRITERIA MINEIGEN(1) ITERATE(25)

/EXTRACTION PC

/CRITERIA ITERATE(25)

/ROTATION VARIMAX

/METHOD=CORRELATION.

**Finally, we performed a principal components regression between Daphnia body size and the retained principal components.**

REGRESSION

/MISSING LISTWISE

/STATISTICS COEFF OUTS R ANOVA

/CRITERIA=PIN(.05) POUT(.10)

/NOORIGIN

/DEPENDENT D.pulicarialength

/METHOD=ENTER FAC1_1 FAC2_1 FAC3_1 FAC4_1.

**3. Analyses of life history traits from the common garden experiments.**

**Age at maturation:**

MIXED ln_age BY Generation Lake Clone Treatment Status

/CRITERIA=CIN(95) MXITER(100) MXSTEP(10) SCORING(1) SINGULAR(0.000000000001) HCONVERGE(0,

ABSOLUTE) LCONVERGE(0, ABSOLUTE) PCONVERGE(0.000001, ABSOLUTE)

/FIXED=Generation Treatment Status Generation*Treatment Generation*Status Treatment*Status

Generation*Treatment*Status | SSTYPE(3)

/METHOD=REML

/PRINT=TESTCOV

/RANDOM=Clone(Lake) Lake(Status) | COVTYPE(VC)

/EMMEANS=TABLES(Status) .

**Clutch size:**

MIXED sq_cl4 BY Generation Lake Clone Treatment Status

/CRITERIA=CIN(95) MXITER(100) MXSTEP(10) SCORING(1) SINGULAR(0.000000000001) HCONVERGE(0,

ABSOLUTE) LCONVERGE(0, ABSOLUTE) PCONVERGE(0.000001, ABSOLUTE)

/FIXED=Generation Treatment Status Generation*Treatment Generation*Status Treatment*Status

Generation*Treatment*Status | SSTYPE(3)

/METHOD=REML

/PRINT=TESTCOV

/RANDOM=Clone(Lake) Lake(Status) | COVTYPE(VC)

/EMMEANS=TABLES(Status) .

**Size at maturation:**

MIXED body_Feret BY Generation Lake Status Treatment Clone

/CRITERIA=CIN(95) MXITER(100) MXSTEP(10) SCORING(1) SINGULAR(0.000000000001) HCONVERGE(0,

ABSOLUTE) LCONVERGE(0, ABSOLUTE) PCONVERGE(0.000001, ABSOLUTE)

/FIXED=Generation Status Treatment Generation*Status Generation*Treatment Status*Treatment

Generation*Status*Treatment | SSTYPE(3)

/METHOD=REML

/PRINT=TESTCOV

/RANDOM=Clone(Lake) Lake(Status) | COVTYPE(VC)

/EMMEANS=TABLES(Status) .

**4. Analysis to test for differences in population growth between the pre- and post-invasion time period from the field-based data.**

UNIANOVA rtwopar BY Lake Status WITH startdensity startdate

/METHOD=SSTYPE(3)

/INTERCEPT=INCLUDE

/CRITERIA=ALPHA(0.05)

/DESIGN=startdensity startdate Lake Status Lake*Status.
